# Chiral Capillary Electrokinetic Chromatography: Principle and Applications, Detection and Identification, Design of Experiment, and Exploration of Chiral Recognition Using Molecular Modeling

**DOI:** 10.3390/molecules26102841

**Published:** 2021-05-11

**Authors:** Sami El Deeb, Camilla Fonseca Silva, Clebio Soares Nascimento Junior, Rasha Sayed Hanafi, Keyller Bastos Borges

**Affiliations:** 1 Institute of Medicinal and Pharmaceutical Chemistry, Technische Universität Braunschweig, 38106 Braunschweig, Germany; 2 Departamento de Ciências Naturais, Campus Dom Bosco, Universidade Federal de São João del-Rei (UFSJ), Praça Dom Helvécio 74, Fábricas, São João del-Rei 36301-160, Minas Gerais, Brazil; camillafonsecasilva@hotmail.com (C.F.S.); clebio@ufsj.edu.br (C.S.N.J.); keyller@ufsj.edu.br (K.B.B.); 3 Department of Pharmaceutical Chemistry, Faculty of Pharmacy and Biotechnology, German University in Cairo, Cairo 11835, Egypt; rasha.hanafi@guc.edu.eg

**Keywords:** chiral capillary electrokinetic chromatography, enantioseparation, chiral selector, capillary electrophoresis, Quality by Design, enantiomers, capillary electrophoresis-mass spectrometry, enantiomeric impurity, molecular modeling, chiral recognition

## Abstract

This work reviews the literature of chiral capillary electrokinetic chromatography from January 2016 to March 2021. This is done to explore the state-of-the-art approach and recent developments carried out in this field. The separation principle of the technique is described and supported with simple graphical illustrations, showing migration under normal and reversed polarity modes of the separation voltage. The most relevant applications of the technique for enantioseparation of drugs and other enantiomeric molecules in different fields using chiral selectors in single, dual, or multiple systems are highlighted. Measures to improve the detection sensitivity of chiral capillary electrokinetic chromatography with UV detector are discussed, and the alternative aspects are explored, besides special emphases to hyphenation compatibility to mass spectrometry. Partial filling and counter migration techniques are described. Indirect identification of the separated enantiomers and the determination of enantiomeric migration order are mentioned. The application of Quality by Design principles to facilitate method development, optimization, and validation is presented. The elucidation and explanation of chiral recognition in molecular bases are discussed with special focus on the role of molecular modeling.

## 1. Introduction

Chirality plays a crucial role in life with a multidisciplinary interest—including, but not limited to, chemistry, biochemistry, food chemistry, agrochemistry, drug discovery, pharmacology, engineering, physics, and material science. Stemming from the chirality of natural amino acids, stereospecificity is a characteristic of many physiological reactions and pharmacological processes for all living systems [1,2,3,4].

Methods for enantioseparation, particularly on an analytical scale, are still gaining attraction, due to the continuous need for chiral separation and determination in different research and industrial fields. Enantiomers that have identical physical and chemical properties, might produce different pharmacological responses. Some counter enantiomers are recognized as being pharmacologically same active, less active, different active, inactive, antagonistic, or even toxic compared to their active enantiomeric pair. Distomer is considered the less potent, while the eutomer is more powerful for a particular action [5,6,7,8,9,10,11].

Even when only considering the presence of inactive counter enantiomer of a drug in racemate, it constitutes an additional load on the body without benefit. Therefore, the number of drugs that are produced as enantiomers continually increases the expense of racemate drugs. Thus, there is usually a need for enantioselective methods to separate and quantitate enantiomers, but also to determine enantiomeric impurities with high sensitivity, which must be in accordance with the International Conference on Harmonization (ICH) regulations to quantify enantiomeric impurity of 0.1% relative to the main enantiomer. This has been established in the guidelines of ICH and the other regulatory authorities, such as the Food and Drug Administration (FDA) and European Medical Agency (EMA), for the validation of analytical methods [12].

Possible racemization of a single enantiomeric drug might occur during synthesis, storage, or in vivo metabolism. Possible in vivo inversion of a single enantiomeric drug (eutomer) to produce the unwanted enantiomer (distomer) in the biological system should be considered early during drug development. In vivo metabolism studies in rats are usually conducted using a proper enantioseparation method [13]. Furthermore, the determination of drug enantiomer, or its metabolite from biological body fluid and tissue samples, is highly valuable to study for its stereoselective pharmacokinetic and pharmacodynamic properties, to understand their enantioselective drug action in addition to stereoselective drug interaction and toxicity [6,14].

Enantioseparation methods are also important in food analysis of protein amino acids, organic acids, and sugars. For instance, it is necessary to check the absence of D-amino acids in food proteins, which might be formed due to racemization of natural L-amino acids during food production technology (e.g., treatment by alkaline conditions or heat). The presence of D-amino acids will decrease the susceptibility of the proteins to proteolytic enzymes [15,16].

Enantiomeric analytical methods are also required for agrochemical analysis of chiral pesticides and insecticides [17]. Environmental analysis of contaminated water (e.g., pharmaceutical wastewater), soil, and food by residues of chiral drug and/or their metabolites and chiral pesticides is an important field. Enantioselective separation is mandatory to explore the potential of these substances to exhibit stereoselective toxicity or degradation and its consequences on animals and humans [18,19]. Enantioseparation is also important in doping and forensic science.

Capillary electrophoresis (CE) and capillary electrochromatography (CEC) play more competitive and complementary roles to liquid chromatography (LC) than the other techniques for chiral separation, such as gas chromatography (GC) and supercritical fluid chromatography (SFC), and to less extent thin layer chromatography (TLC) [6,20,21,22,23,24,25]. Microchips also contribute to the field of enantiomeric separation and detection with successful applications [26].

Compared to LC, the application of CE for chiral analysis involves lower sample and reagent consumption, making it a green alternative enantioseparation technique. CE techniques are also known to offer higher separation efficiency and resolution and mostly lower analysis time than HPLC. However, LC remains superior with regard to UV detection sensitivity and also mostly with regard to the repeatability of migration time and peak area. Moreover, the chiral separation principle in CE is different from that in HPLC, as described below. Therefore, CE is a good complementary technique to HPLC for chiral separation. It is also worth noting that most successful chiral selectors in HPLC can be transferred to CE except for aqueous buffer insoluble ones [27].

Among other CE modes, electrokinetic chromatography (EKC) is a widely used CE mode for enantioseparation [6,28,29]. EKC modes include micellar electrokinetic chromatography (MEKC), in which a micelle is used with or without chiral selector (CS) as a pesudostationary phase (PSP), and microemulsion electrokinetic chromatography (MEEKC), in which a microemulsion is used as carrier electrolyte. The present review draws attention to nonmicellar chiral capillary electrokinetic chromatography (CEKC) as a well-established technique for enantioseparation. The use of chiral MEKC is limited because uncharged enantiomeric compounds can also be separated by nonmicellar CEKC with the charged chiral selector. MEKC and MEEKC are out of the scope of this review and are described elsewhere [30,31,32,33,34,35,36].

This review gives an overview of the development and application of CEKC from January 2016 to March 2021. Methodological and application advances, for the use of the technique in the enantiomeric separation and enantiomeric impurity determination, are discussed, and new chiral selectors are reported. In addition, the successful use of Quality by Design is explored, and the application to elucidate the mechanism of enantiomeric separation and the enantiomer migration order (EMO) is critically reviewed with special highlights on challenges, solutions, and future aspects.

## 2. Chiral Capillary Electrokinetic Chromatography Principle and Selectors

### 2.1. Principle of CEKC

CEKC is a CE mode in which a CS is added directly to the background electrolyte (BGE). The separation of molecules will then result from the dynamic equilibrium with the CS inside the capillary and their different electrophoretic mobilities, if present, in a charged form in the BGE [37,38].

The added CS to the BGE will form noncovalent transient diastereomeric complexes with each of the two enantiomers of mostly the same drug with different complexation constants, and thus, different complex stability. In addition to the nonstereospecific binding forces, each enantiomer will form additional stereospecific binding with the chiral selector, mostly hydrogen bonding, but also forces like hydrophobic, electrostatic, van der Walls, and steric factors. In addition to possible inclusion forces, if cavity (or more) is/are available within the structure of the CS, then part of the enantiomer can pass inside. The two enantiomers will mostly have different affinity to the CS, i.e., the enantioseparation can be reached by different intermolecular forces and thermodynamic selectivity in the recognition mechanism [39,40,41].

It is proven, however, that it is possible in CE, and not in HPLC, that the enantioseparation selectivity can exceed the thermodynamic selectivity of the recognition. Therefore, CE can separate an enantiomeric pair of a compound even if the binding constants of its individual enantiomers with the CS are identical. That is to say, both or either a binding constant difference or analyte difference is/are required to achieve enantioseparation in CEKC [27,42].

Therefore, each enantiomer will migrate with a different speed (electrophoretic migration) because of the different interactions with the chiral selector, and thus, the two enantiomers will separate from one another. High flexibility is offered in choosing one (or more) of a variety of CS available for enantioseparation and simply adding it/them to the BGE in combination or not. This also offers the ability to easily adjusting the concentration of the CS to achieve the best equilibrium and to modify the electroosmotic flow (EOF) velocity through an effect on viscosity for best enantioseparation [43,44].

The CS can easily and cost-effectively be replaced by another in CEKC to identify the one with the best separation power for the intended enantiomeric molecules or to invert the migration order of the enantiomer peak pair. The concentration of the CS can also be modified for best selectivity [21,28].

**Figure 1:**
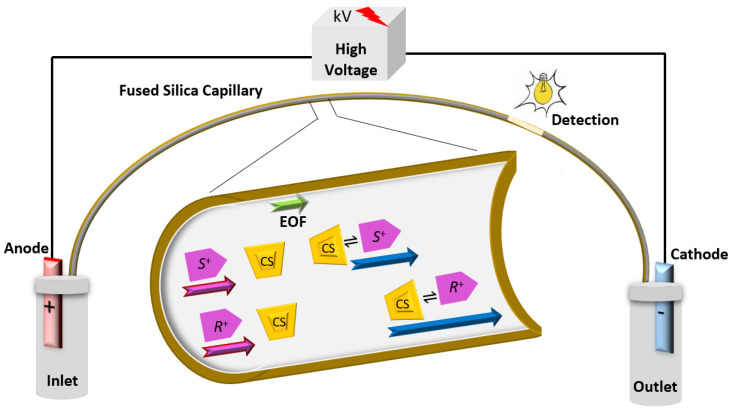
Simple representative schematic diagram of CEKC normal polarity mode of the separation voltage separating a basic ionized enantiomeric racemate (*R*^+^ and *S*^+^) using CS in the BGE. Pink and green arrows show the apparent mobility (µ_app_) of the enantiomeric pair and the mobility of the EOF (µ_EOF_), respectively. The blue arrows show the difference in the speed of the migration of enantiomers upon interaction with the CS. µ_app_ of the CS would be according to its charge.

**Figure 2:**
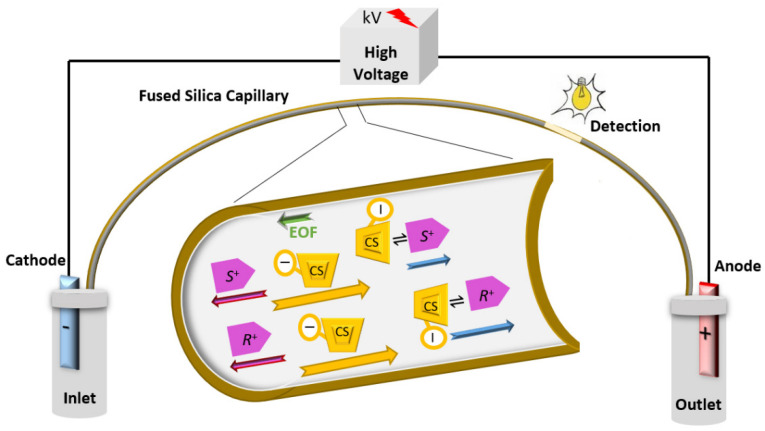
Simple representative schematic diagram of CEKC under reversed polarity mode of the separation voltage with a negatively charged chiral selector and a basic ionized enantiomeric pair (*R*^+^ and *S*^+^). Pink and green arrows show the apparent mobility (µ_app_) of the enantiomeric pair and the mobility of the EOF (µ_EOF_), respectively. The blue arrows show the difference in the speed of the migration of enantiomers upon interaction with the CS. Complexed enantiomers will migrate in the direction of the anode (here the outlet), due to interaction with the negatively charged CSs, which have strong µ_app_ in the direction of anode.

CEKC can be conducted under normal or reversed polarity modes of the separation voltage. Enantiomers will migrate in a direction depending on its apparent mobility (µ_app_), which is the sum of the electrophoretic mobility (µ_e_) and the EOF mobility (µ_EOF_), but also influenced by the µ_app_ of the CS to which it will complex. The normal polarity of the separation voltage (Figure 1) indicates having the anode (+) at the inlet and the cathode (−) at the outlet. Under the normal polarity mode of the separation voltage, the EOF is towards the cathode (detector/outlet). The normal polarity mode of the separation voltage is the standard mode in CE. In contrast, the separation can be conducted under a reversed polarity mode of the separation voltage. The reversed polarity mode of the separation voltage indicates having the cathode at the inlet and the anode at the outlet. Under reversed polarity of the separation voltage, the direction of the EOF will be mostly away from the detector, except if a zero-flow capillary or a low pH BGE is used. Negatively charged enantiomers with electrophoretic mobility greater than the EOF will pass the detector. Negatively charged cyclodextrins (CDs) have strong electrophoretic mobility toward the positive electrode (anode). Basic drugs are positively charged under low pH buffer and are more likely to interact with negatively charged CD, and then the CD drug complex will be attracted toward the anode at the outlet and pass the detection window (Figure 2). Therefore, the negatively charged CDs will act as a carrier of enantiomers by their self-mobility toward the anode at the outlet [36,45,46,47,48].

### 2.2. Types of Chiral Selectors in CEKC

Over the past years, many substances have been successfully used or unsuccessfully tried (e.g., without achieving the required resolution) as chiral BGE additives in nonmicellar CEKC to separate enantiomeric pairs. In principle, any substance with potential enantiomeric separation power, good stability in the BGE, and compatibility with the detection mode can be tried. Recently, new substances are showing good ability to separate enantiomers [49,50]. Additional special considerations should be taken, for example, when using EKC-mass spectrometry (MS), as described in Section 3.1.

**Figure 3:**
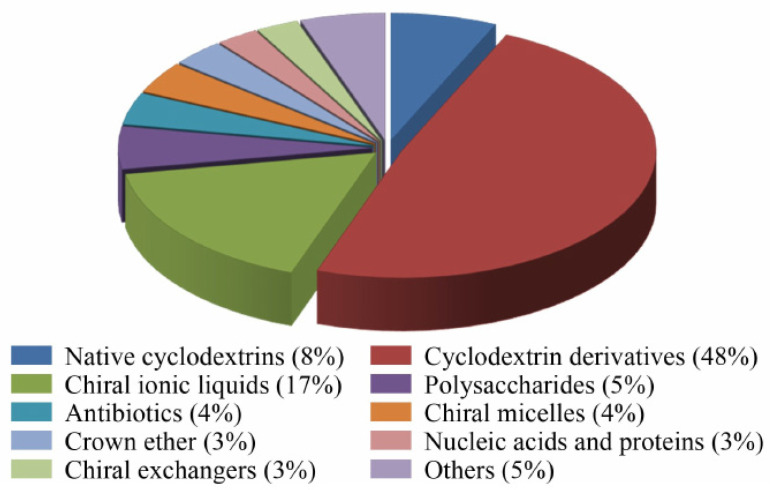
The proportion of application of the various chiral selectors in CE in 2015–2019 (incomplete statistics). Reprinted with permission from reference [51].

In a published work from September 2020 about recent advances of novel chiral separation systems in CE, Qi [51] provided a circular statistical graphic (circle chart) divided into slices to illustrate the numerical proportion of the application of CSs in enantiomeric separation by CE, as shown in Figure 3.

A similar chart with a special focus on CSs in CEKC during 2017–2018 has before been published by Yu and Quirino [28]. Both authors show that derivatized CDs as a single selector are the most commonly used CSs and are responsible for about 50% of chiral separation methods.

Up-to-date derivatized CDs, which are formed through chemical substitutions with a stronger recognition ability and/or better solubility, are still pioneering the field of CSs in CEKC to separate enantiomers in either pharmaceutical formulations, biological samples, or other fields [52].

Uncharged derivatized CDs include substances as hydroxypropyl-α-CD, hydroxypropyl-β-CD, and hydroxypropyl-γ-CD. Negatively charged derivatized include high sulfated-α-CD, high sulfated-β-CD, high sulfated-γ-CD, sulfobutyl-ether-β-CD, carboxymethyl-β-CD, phosphated-α-CD, phosphated-β-CD, phosphated-γ-CD, and succinylated-β-CD. Positively charged derivatized CDs as a quaternary ammonium CD also showed successful applications. Among others, hydroxypropyl derivatized CDs are claimed to have the best enantioseparation power for a wider variety of chiral compounds [6,52,53,54,55].

Negatively charged CDs (anionic CDs) are also especially important and widely applied for the enantioseparation of positively charged cationic compounds (basic chiral compounds) and neutral chiral compounds [56].

The single isomer CDs are preferred for chiral separation to obtain reproducible results, but also to study aspects of the selector-analyte interactions and to better understand the experimental design of chiral CE separations [53].

The CDs have been applied mostly as a single chiral selector, but also sometimes in a dual or multiple chiral system, as described below. Tuning of enantio-discrimination can be achieved by substitution of the single CD molecule, but also fine-tuning is possible by modifying the other separation conditions [53]. Cucinotta et al. [57] synthesized and characterized a new capped derivative of β-CD. The synthesized new lysin-bridged hemispherodextrin was characterized by electrospray ionization mass spectrometry (ESI-MS) and Nuclear magnetic resonance (NMR) spectroscopy. Circular dichroism and electron spin resonance spectroscopy were used to test its inclusion ability and its metal coordination ability, respectively. The new selector has been used to separate enantiomers with improved ability toward anionic and cationic guests compared to the single free CD. Recently, Salido et al. [58] reported the effect of eutectic solvents as choline chloride-ethylene glycol, choline chloride-urea, choline chloride-D-glucose, and choline chloride-D-sorbitol as additives in CD mediated CEKC to improve the enantioseparation of lacosamide enantiomers. The addition of choline chloride-D-sorbitol to the separation media containing succinyl-β-CD has been found to increase the resolution value from 1.5 to 2.8.

Room temperature ionic liquids (ILs) are the second most commonly used CSs in CEKC after CDs. ILs are responsible for about 17% of chiral separation. Chiral ILs (CILs) are organic salts that consist of organic cation and an organic or inorganic anion. Either the cation or the anion must be chiral to consider as CILs [28,59]. Recently, Nie et al. [60] reviewed and categorized ILs for enantioseparation and explored their resolution mechanism. Amino acid-based ILs have been proposed for potential use as sole CSs in CEKC [61]. Zhang et al. [62] reported the potential use of novel mono- and di-tetraalkylammonium L-tartrate ILs with different alkane chain lengths as sole CSs in CE. Successful enantioseparation of five model chiral analytes has been obtained with a high proportion of methanol. In many cases, ILs could not show enough enantiomeric discrimination power when used as sole CSs, and thus, have been usually used in combination with other CSs, mostly CDs, and particularly the derivatized ones. They act in synergism together to achieve better enantioseparation. It is suggested that the mechanism of separation is then based on the competition of the chiral IL and the enantiomer for inclusion into CD [63,64,65,66,67]. Wahl and Holzgrabe prepared and used ILs combining tetrabutylammonium cations with chiral amino acid-based anions (tetrabutylammonium l-argininate) together with β-CD for the enantioseparation of ephedrine, pseudoephedrine, and methylephedrine isomers [68].

Polysaccharides, either the homo-polysaccharides as maltodextrin or the glycosaminoglycan as chondroitin sulfate and heparin, are contributing to about 5% of chiral separation and are much behind CDs in their enantiomeric discrimination power [69,70,71]. Sun et al. [72] described a CEKC approach using ethanediamine-bonded poly (glycidyl methacrylate) microspheres as CSs for enantioseparation with chondroitin sulfate E. Better enantioseparation results were obtained using the microsphere for separating basic model chiral compounds compared with using the single system of chondroitin sulfate E.

Macrocyclic antibiotics have molecular masses range between 600 and 2200. Their enantioseparation power is because they have multiple stereogenic centers. Most are accessible in charged forms, and thus, suit CEKC of uncharged enantiomeric drugs [73]. Examples include eremomycin, rifamycin B, vancomycin, thiostrepton, ristocetin A, fradiomycin, teicoplanin, azithromycin, erythromycin, and clarithromycin. Most of them have several chiral centers with or without cavities (e.g., teicoplanin has 23 chiral centers and 4 cavities), ensuring good enantioseparation power [74]. Post run washing of the capillary and the use of small volume samples have been recommended because of the strong ability of the glycopeptide antibiotics to adsorb to the inner wall of the capillary [75,76].

Recently, Ren et al. [77] reported using the second-generation macrolide antibiotic gamithromycin as a novel chiral selector for enantioseparation in CE. Gamithromycin showed particular enantioseparation power for chiral primary amines.

In another recently published research work, the steroid antibiotic fusidic acid has been proposed as a novel chiral selector for enantioseparation in CE. The authors suggested its special effective enantiodiscrimination power for chiral analytes containing rigid planar structures. The authors expected the finding to open the door for investigating other structurally similar steroids for possible enantioseparation power [78].

Chiral metal-ion complexes are small molecule chiral selectors for CEKC. Example include Cu(II)-L-ornithine, Cu(II)-D-phenylalanine, Cu(II)-l-lysine, Cu(II)-D-quinic acid, Zn(II)-l-arginine, Zn(II)-l-lysine, Co(II)-D-quinic acid and Ni(II)-d-quinic acid. They act through the formation of diastereomeric ternary metal-ligand-analyte complexes. Meta ligand complexes showed successful application for the enantioseparation of amino acids and dansylated amino acids, and glycyl peptides [79,80].

Among proteins, human or bovine serum albumins are the most commonly used chiral selectors in CEKC. A good washing protocol is required to minimize protein adsorption to the inner surface of the fused silica capillary. Proteins are still playing a particularly good role in investigating enantioselective protein interaction with enantiomeric drugs [81]. Ratih et al. [7] used human serum albumin as a chiral selector in 20 mM phosphate buffer pH 7.4 for enantioseparation and simultaneous binding constant determination of amlodipine and verapamil enantiomers. It is worth noting that microscale thermophoresis [82] also is useful in the study of enantioselective affinity of chiral drugs toward therapeutic target proteins [83].

Quintana et al. [84] synthesized carbosilane dendrimers functionalized with L-cysteine and *N*-acetyl-L-cysteine on their surface and used them as CS to separate razoxane by CEKC. Results showed partial resolution, but opens the door for more research in this regard to explore the use of dendrimers as CSs in CEKC.

New selectors are continuously proposed to enhance enantioselectivity. Meanwhile, modifications of existing selectors, for example, by derivatization, also takes place. However, random derivatization processes that cannot identically be repeated to produce the same product are not recommended, even if a good separation is obtained, because no result reproducibility can then be ensured.

**Figure 4:**
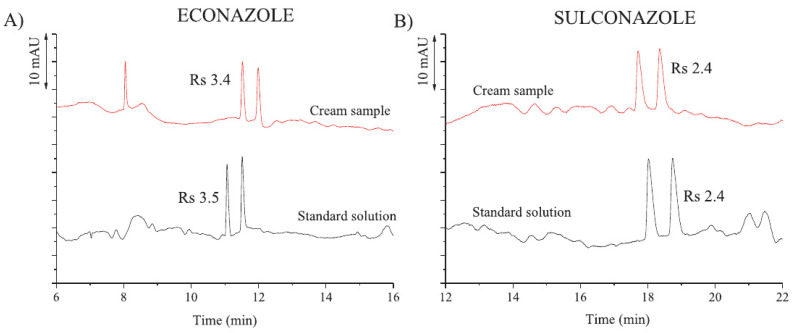
Electropherograms that correspond to the enantiomeric separation of a standard solution (40 mg/mL) and cream samples of econazole and sulconazole. CE conditions: 50 mM phosphate buffer pH 2.5 containing mixtures of (**A**) 5 mM hydroxypropyl-β-CD with 20 mM tetrabutylammonium-L-lysine at 25 °C; (**B**) 2 mM hydroxypropyl-β-CD with 25 mM tetrabutylammonium-L-lysine at 15 °C. Other conditions: Uncoated fused silica capillary, 50 μM i.d. × 48.5 cm (40 cm of effective length); UV detection at 200 nm; applied voltage, 30 kV; injection by pressure, 50 mbar for 10 s. Reprinted with permission from [92].

Combination of two CSs in a dual chiral separation system or more in multiple chiral separation systems have been reported to improve the enantioseparation [85,86]. A dual chiral separation system is less complicated than the multiple ones, and is more commonly used. This has been achieved mostly by adding the two CSs mixed together to the BGE or sometimes by injecting them separately in two separated plugs without meeting each other in the capillary. Many examples showed an enhanced separation efficiency through the synergetic effect of dual CS systems of two mixed chiral selectors [87]. A dual CDs CS system of sulfobutyl-ether-β-CD and native γ-CD resulted in a good enantioseparation of lansoprazole and rabeprazole [88]. Nicolaou et al. [89] developed two dual CS systems to separate the enantiomers of warfarin, coumachlor, nefopam, and fexofenadine using either CD or cyclofructan with the CIL L-alanine tert butyl ester lactate. The addition of the CIL to the BGE was found to improve the resolution. Six basic racemic drugs (amlodipine, chlorphenamine, duloxetine, propranolol, nefopam, and citalopram) were separated using a dual chiral selector system of chondroitin sulfate D and carboxymethyl-β-CD [90]. Chen et al. [91] reported the synergetic action of the IL tetramethylammonium-L-arginine and maltodextrin in enantioseparation of the five studied drugs, including nefopam, duloxetine, ketoconazole, cetirizine, and citalopram. Better resolution values were obtained compared to the single maltodextrin system. Salido-Fortuna et al. [92] reported a CEKC method for the enantiomeric determination of econazole and sulconazole in pharmaceutical formulation using a dual chiral system consisting of hydroxypropyl-β-CD combined with ionic liquids. Best separation was obtained with hydroxypropyl-β-CD combined with tetrabutylammonium-L-lysine. High enantiomeric resolution values for econazole and for sulconazole have been achieved, as shown in Figure 4.

Some examples showed an enhanced separation efficiency through dual CS systems in which the two chiral selectors are introduced to the capillary in two separated plugs. Chalavi et al. [93] developed an enantioseparation method based on the partial filling technique with two chiral selector plugs. The first plug contains either hydroxypropyl-α or β-CD and the second plug contains maltodextrin. The two adjacent chiral plugs contain the same BGE. Each plug is supposed to separate the enantiomers independently. This approach is aimed to prevent the possible unwanted interaction between the two chiral selectors inside the capillary and is useful for chiral compounds that cannot be separated using either one of the two intended chiral selectors or a mixture of them. The method showed success in separating racemic drugs, including baclofen, carvedilol, cetirizine, chlorpheniramine, citalopram, fluoxetine, hydroxyzine, propranolol, tramadol, and trihexyphenidyl. No significant effect has been noted in selectivity factors (α) and migration times, and no significant differences between the resolutions when the order of the two chiral selector plugs in the capillary has been inverted. One should, however, note that in other cases (e.g., CDs with ILs), the interaction between the two chiral selectors is mostly advantageous and wanted to produce a synergetic effect.

Recently, La et al. [86] developed a CEKC method based on a multiple chiral separation system. The method has been used for separating dihydropyridone analog, a drug candidate proposed in type 2 diabetes treatment, which is a neutral hydrophobic molecule with multiple chiral centers. Its eight isomers have been successfully separated using a triple CD system consisting of 15mM sulfobutyl-ether-β-CD plus 15mM γ-CD and 40 mM hydroxypropyl-γ-CD in a 50 mM borate BGE at pH 10. Further relevant applications of CEKC dating between January 2016 and March 2021, other than those described in the text of this review, are summarized in Table 1.

## 3. Methodological and Instrumental Aspects

### 3.1. Detection Sensitivity

One main drawback of CE-UV is the lower concentration sensitivity compared to LC-UV, which also applies in CEKC-UV, especially when intended to be used for enantiomeric impurity quantitation from a pharmaceutical preparation or enantiomeric determination from a biological sample. This stems from the lower injection volume in CE compared to HPLC in addition to the short optical path-length of the capillary (capillaries are used with diameters ranging between 25 to 100 µm, usually 50 µm). Therefore, a number of in-capillary preconcentration techniques can be applied to increase the sensitivity of a UV detector [113,114,115]. The use of different capillary forms with a longer path-length at the detection window (e.g., bubble cell, bent cell, or multireflection capillaries) is also an alternative to increase sensitivity.

It is important to note that two separated enantiomers may path the detection window of a capillary connected to the detector with different speeds, the difference in mobility residence in the detection window can create a difference in detection response between the two enantiomers. This is particularly important to aware of when checking impurity enantiomer percentage as %area/area.

In case of very low sensitivity detection or for non-UV-absorbing enantiomeric compounds lacking the chromophoric group, using other detection techniques as, for example, electrochemical or conductivity detection or mostly mass spectrometry is recommended [116]. Derivatization can be performed to enhance detection. Wuethrich and Quirino reviewed the four possible modes of derivatization for CE; precapillary, in-line, in-capillary, and postcapillary derivatization [117]. The precapillary derivatization with 9-fluorenylmethyl chloroformate and subsequent UV-detection or MS detection is an option for some compounds [118,119]. Laser-induced fluorescence (LIF) detection did not show popularity in CEKC, due to the drawback of the molecular labeling used for enantiomeric drug derivatization. Degradation products of the fluorophores can generate fluorescent side products, which can interfere with the detection signal of the labeled low concentration target enantiomer [120,121].

Alternatively, high sensitivity detection techniques as MS can be used. For enantiomeric determination from biological samples with a complex matrix and usually a low concentration of the target enantiomeric substances and/or its enantiomeric metabolites, initial sample treatment is required via the use of a sample preconcentration technique.

The sample preconcentration is aimed to clean up the sample from the complex matrix by extracting the target enantiomer, but also concentrating it to increase its detectability. The most commonly used sample preconcentration technique is solid phase extraction (SPE) [94,105].

SPE with different stationary phases as per target analyte, including, e.g., anion exchange or cation exchange cartridges, is the most commonly used sample preconcentration technique. SPE cartridges for almost every kind of analytes are nowadays commercially available. SPE process can simply be performed off-line, e.g., for research purpose, but also on-line SPE is available for large scale analysis. Besides SPE, other preconcentration techniques as liquid-liquid extraction (LLE), liquid-liquid microextraction (LLME), pressurized liquid extraction (PLE), dispersive liquid-liquid microextraction (DLLME), electromembrane extraction (EME) have also been reported in enantiomeric separation and quantitation by CE. Evaluation of each depends on extraction efficiency and recovery percentage in addition to simplicity, speed, and cost-effectiveness [6]. It is worth noting that subsequent on-line sample preconcentration techniques to focus the analyte on-capillary before its detection as sweeping and stacking at the interface between the sample and BGE zones to improve the detectability can be applied [114,115,122].

Hyphenation to MS offers higher sensitivity and selectivity and provides structural information, which might be needed, especially when dealing with chiral metabolites. Improved limits of detection (LODs) are usually obtained with CEKC-MS compared to CEKC-UV. For CEKC-MS hyphenation, ammonium-based volatile buffer components should replace the classical nonvolatile buffer components of the BGE as phosphate and borate buffers. The coupling of CEKC to MS is still not fully straightforward. The possibility of the nonvolatile chiral (low molecular-weight) CSs to either induce ion suppression and interfere with the MS signal in the m/z range of the enantiomeric analyte or induce source contamination of the MS ion source and optics, thus decrease the sensitivity should be considered. In this regard, a lower concentration of the selector or a slower EOF can be considered to decrease the entry of the chiral selector to the ion source. It is worth noting that a successful CEKC-MS has been reported using native β-CD with a moderately tolerable ionization suppression allowing smooth coupling to MS [95,123].

Compared to chiral MEKC, the absence of surfactant in nonmicellar CEKC make it more suitable for coupling with MS. However, in MEKC, a number of high molecular mass polymeric chiral surfactants have been used as polymeric micelles to replace the conventional surfactants forming micelles. They are claimed to remain intact and not fragment in the gas phase of the mass spectrometer, thus offering better compatibility of MEKC with MS by avoiding forming background ions from fragmented conventional surfactants into surfactant monomers. Moreover, volatile PSPs have also been used in chiral MEKC indirect mode based on derivatization of the analyte [30,124]. Likewise, stable crown-ether complexes in CEKC are used without a problem when coupled with MS [125].

**Figure 5:**
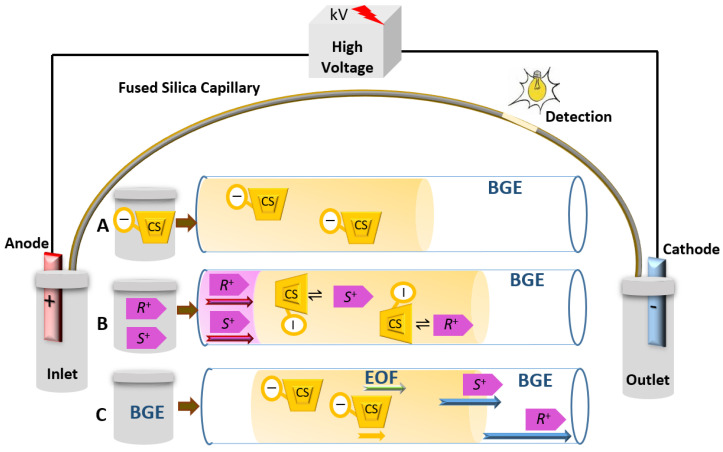
Representative schematic illustration of enantioseparation by CEKC applying PFT for separating a basic ionized enantiomeric pair (*R*^+^ and *S*^+^) using CS in the BGE. Filling the CS (A), injecting the enantiomeric compound (B), and conducting the run with a vial contain BGE (C). Pink, yellow and green arrows show the apparent mobility (µ_app_) of the enantiomeric pair, the CS, and the mobility of the EOF (µ_EOF_), respectively. The µ_app_ of the CS is also in the direction of the cathode, but the migration speed of the CS is very slow compared to the migration speed of the two enantiomers; thus, the enantiomers can pass the CS plug rapidly. The blue arrows show the difference in the speed of the migration of enantiomers upon interaction with the CS.

Two filling techniques are available to introduce the chiral selector into the BGE inside the capillary with a selector-free detection window. The first is the partial filling technique (PFT). PFT is an alternative CS filling technique to the complete filling technique (CFT). CFT involves filling the capillary with CS, and then injecting the chiral compound, and doing the CE run from a solution of chiral selector in the running buffer [29,126]. As an alternative, the separation could be conducted by PFT, in which the CS is introduced into the capillary from a solution of the CS in the buffer, and then the chiral compound is injected. The run is subsequently conducted from a vial containing only the buffer without the CS (Figure 5) [127,128].

PFT reduces the consumption of the chiral selector. The technique works best if the enantiomers have apparent mobility higher than EOF mobility to be able to pass the chiral selector plug in the capillary. Otherwise, a low pH buffer is used to decrease EOF mobility, or alternatively, a coated capillary is recommended [29]. The PFT is claimed to counter the background absorbance when a UV detector is used and is more suitable to CEKC-MS to avoid (or minimize) the entry of CS to the ion source [29,129].

Lee et al. [130] developed a CEKC-MS enantioseparation method for underivatized free amino acids (AAs) using a PFT for the determination of D-AAs in vinegar. Crown ether (18-crown-6)-2,3,11,12-tetracarboxylic acid (18C6H4) was used as chiral selector. The method resulted in the simultaneous separation of seventeen AAs.

**Figure 6:**
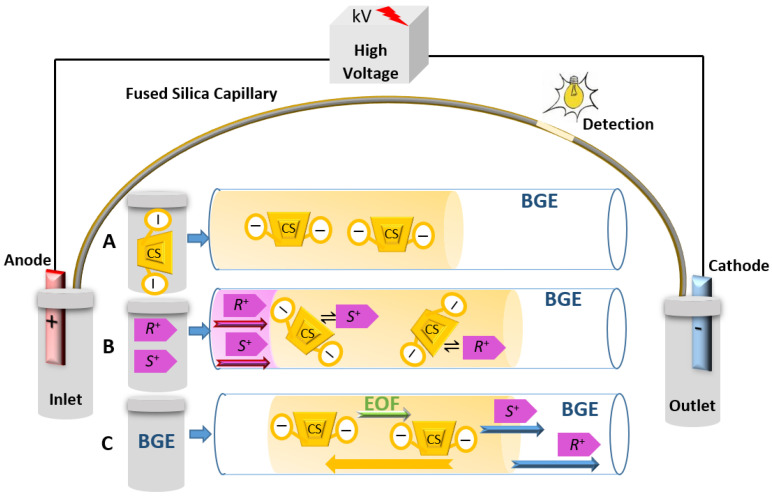
Representative schematic illustration of enantioseparation by CEKC applying counter migration technique for separating basic ionized enantiomeric pair (*R*^+^ and *S*^+^) using CS in the BGE. Filling the CS (A), injecting the enantiomeric compound (B), and conducting the run with a vial contain BGE (C). Pink, yellow and green arrows show the apparent mobility (µ_app_) of the enantiomeric pair, the CS, and the mobility of the EOF (µ_EOF_), respectively. The CSs will have µ_app_ toward the anode and will migrate to the opposite direction of the detector. The blue arrows show the difference in the speed of the migration of enantiomers upon interaction with the CS.

The second technique to minimize the entry of the selector to the ion source is the use of the counter migration technique, in which the CS will have strong electrophoretic mobility to the anode (stronger than the EOF) away from the cathodic end and the detector (Figure 6). Therefore, the CS is supposed not to inter the ion source when the CEKC is hyphenated with MS. Highly negatively charged derivatized CDs like sulfated-β-CDs can experience this phenomenon [29,131].

Successful CEKC-MS methods have been reported using different ion sources, while ESI is the most commonly used ion type. In one study, CEKC-MS was used for simultaneous enantioseparation and determination of a number of amphetamine-type stimulants and ephedrine using sulfated-γ-CD as CS, which achieved LODs in order µg/mL [132]. In another study, CEKC coupled to inductively coupled plasma-mass spectrometry (ICP-MS) was applied for the enantioseparation and determination of oxaliplatin enantiomers using sulfated-β-CD as CS. LOD and limit of quantification (LOQ) were 64 ng/mL and 116 ng/mL, respectively [133]. The CEKC-MS/MS method has also been reported with further improvement in sensitivity, due to the reduction of noise, thus increase in S/N ratio, and the further improvement in selectivity, due to more fragmentation of ions to provide better information about the enantiomer or its metabolite [134,135].

### 3.2. Identification of Individual Enantiomers

**Figure 7:**
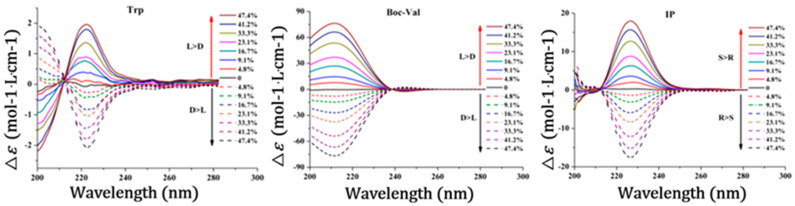
ECD spectra of tryptophan (Trp), N-(tert-Butoxycarbonyl)-valine (Boc-Val), and ibuprofen (IP) with different enantiomeric excess value (ee% value). Enantiomers of each compound have opposite signals. Reprinted with permission from reference [138].

The identification of individual enantiomers is highly valuable for the quantitation of impurity enantiomers, and to follow up a stereoselective synthesis procedure or to determine a stereoselective metabolite [136]. The identification of individual enantiomers of a racemate after enantiomeric separation can directly be achieved by running a reference optically pure single enantiomer under the same electrophoretic conditions and comparing the obtained migration time of the peak with the migration times of the two peaks of the separated enantiomers (peak matching). Alternatively, the peak spiking approach to the racemate sample is also a common method. When the individual enantiomers are unavailable, several alternative approaches are used with different degrees of success and limitations. The polarimetry or electronic circular dichroism can be used to identify the individual enantiomers [137,138]. Enantiomers will have mirrored circular dichroism spectra, which would be equal but opposite. Liu et al. [138] described a CE method in combination with circular dichroism spectroscopy for the identification of enantiomeric peaks in the absence of a single enantiomer standard. Seven enantiomeric substances (tryptophan, tyrosine, phenylalanine, Boc-valine, Boc-leucine, ibuprofen, and naproxen) were used in the study. The method was found to be more suitable for a nonracemic mixture with good and stable electronic circular dichroism signals. Figure 7 shows ECD spectra for typtophan, *N*-(tert-Butoxycarbonyl)-valine and ibuprofen enantiomers.

**Figure 8:**
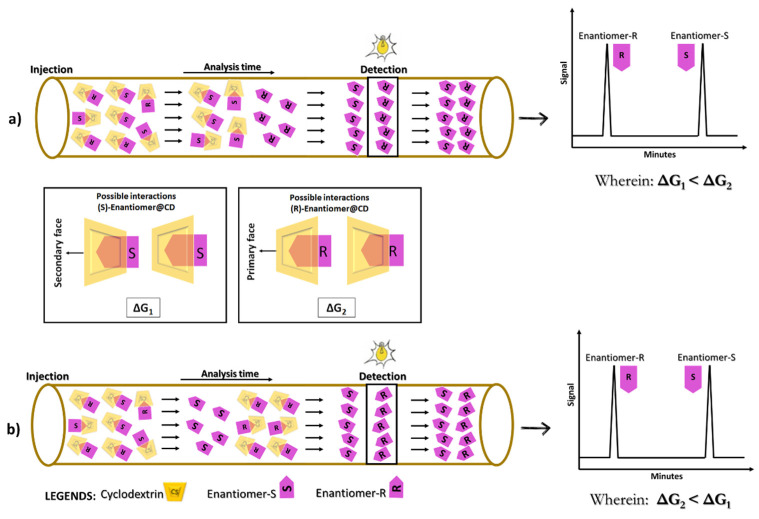
Schematic representation of the chiral discrimination process by CDs. In (**a**), a system is shown in which the CDs migrate in the opposite direction to the EOF, so that the enantiomer involved in the more stable complex ((S)-Enantiomer@CD, correlated with ΔG1), has a longer time of migration. In (**b**), a system is shown in which the CDs migrate in the same direction as the EOF, so that the enantiomer involved in the more stable complex ((R)-Enantiomer@CD, correlated with ΔG2), presents a shorter migration time.

Computational docking studies can give poor information (not accurate) when the structure of the chiral selector is available depending on the different strengths of complexation (discussed below). If the chiral selector is negatively charged and migrates against the EOF and the enantiomer is positively charged, then the enantiomer with the low interaction with the complex is the one that will appear first in the electropherogram with a shorter migration time [44]. For example, for CDs that migrate in the opposite direction to the EOF, it is expected the enantiomer involved in forming the more stable complex will present a longer migration time in the electrophoretic system, reaching the detector last. However, if the chiral selector migrates in the same direction as the EOF, it is expected the enantiomer involved in forming the more stable complex, presumably, presents the shortest migration time, reaching the detector first [139]. Figure 8 schematically illustrates the chiral discrimination process, assuming the influence of EOF and the migration direction of the CDs.

For enantiomeric impurity determination, e.g., from an enantiopure pharmaceutical formulation (supposed to contain pure enantiomeric drug), the relative concentration sensitivity seems to be more relevant than the absolute concentration sensitivity [27]. According to ICH guidelines, the enantiomeric impurity should not exceed 0.1% relative to the main enantiomer. The determination of impurity enantiomer of 0.1% or less in the presence of the main enantiomer in 99.9% makes the separation factor more critical than the absolute concentration sensitivity. It is worth noting that the EMO plays an important role in this regard, and an impurity enantiomer peak eluted before the peak of the main enantiomer is less likely to overlap with the possible tailing effect of the main enantiomeric peak.

Sometimes reversal of the EMO (e.g., by using another chiral selector) might be recommended to ensure better resolution. While also important in this regard, absolute concentration sensitivity plays a more crucial role in determination from biological samples where the enantiomeric pair or their metabolites are present in very low concentration. It is worth noting that most commercially available single pure enantiomeric reference materials contain a certain percentage of the other enantiomer as an impurity. This should be considered when using the reference enantiomer for the quality control of drugs marketed as pure enantiomers [140].

### 3.3. Design of Experiment

In the last decades, pharmaceutical regulatory authorities and organizations have been demanding implementation of a risk-based approach during analytical method development to assure the quality of experimental designs adopted to reach the optimum analytical conditions prior to wet lab development, in addition to delimiting the Design Space and robust areas of an analytical method before proceeding to its method validation. Notions like Analytical Quality by Design (AQbD) were introduced to represent the analytical façade of the general Quality by Design (QbD) concept that was primarily innovated for industrial production before it extrapolates to all fields related to pharmaceutical development. In CEKC, AQbD started replacing Quality by Testing in the last decade, not only to find optimum values of the Critical Method Parameters (CMPs) within the Design Space (DS) (also known as Method Operability Design Region, Knowledge Space, Response Surface, Resolution Map, Contour Plot and Surface Plot), but also to enable a deep understanding of the interactions taking place between analytes enantiomers and the CSto reveal dynamics of the enantiorecognition mechanisms (supported by computer modeling), enhance gained knowledge and risk management of the method that allows enlightened continuous method improvement. Design of Experiments (DoE) has been adopted as the systematic approach to assure the quality of the experimental design. In CEKC, DoE starts by screening or scouting CMPs (numerical, such as voltage and temperature, and categorical, such as nature of chiral selector, BGE, etc.) that significantly affect the Critical Quality Attributes (CQAs) that are predominantly enantioresolution and total run time where the highest resolution and lowest migration time are usually the intended Analytical Target Profile (ATP). The most common designs during this screening phase are Fractional Factorial and Placket Burman designs, yet some reports overlook a multivariate screening and resort to the more time-consuming One-factor-at-a-time (OFAT) (Table 2). Settling on the most relevant CMPs is usually aided by Main Effect Plots and Pareto charts, however none of the methods reported in the interval January 2016-March 2021 invested effort in this phase where many used the OFAT approach, while others did not proceed via the statistical analysis step (Table 1), probably due to the established CMPs in CEKC, namely, voltage, temperature, nature and concentration of chiral modifier and buffer in the BGE. In a study by Milan et al. [110], nine types of CDs were screened at four different pH values using OFAT, meaning 36 runs, which has certainly consumed an enormous time, while a nine-factor Placket Burman screening design would have needed only eight runs and proper statistical interpretation using Main Effect Plots and Pareto charts.

All computation was indeed focused on the optimization phase, where designs like full factorial, face-centered central composite designs, D-optimal designs are adopted to produce the response surfaces and generate the multivariate or least-square regression models related the significant CMPs to CMAs in addition to Monte Carlo simulations that are used for accurate definition of the DS, that enables selection of the optimal working point, robust range, or sweet spot revealed by the overlay of multiple methods operable design region (MODR). Responses in multivariate optimization in CEKC are mostly selectivity or resolution, and analysis time, where their simultaneous optimization is sometimes performed via the desirability function [110,141].

Selection of the appropriate optimization method is critical to the accuracy of fit of the regression model obtained. Long expertise of the analyst sometimes allows him to skip this step where a selection of relevant factors to carry to the optimization design is an expert decision; sometimes, classic trends between CMPs and CQAs like the direct relation of run time with buffer concentration and its inverse relation with voltage can lead to skipping those two factors in a screening study. However, there is no guarantee then of the appropriateness of model fit that is hypothesized to be: Y = β0+ β1 × 1+ β2X2+ β3X3+ β4X4+ β11X12+ β22X22+ β33X32+ β44X42+ β12X1X2+ β13X1X3+ β14X1X4+ β23X2X3+ β24X2X4+ β34X3X4+ ε, where Y is the CQA, X are CMPs with different orders and two-way interactions, and ε the random error.

In a study by Abdel-Megied et al. [141], a screening design was missing, and a full factorial model of two factors at three levels was erroneously selected to optimize the effect of CD and pH, where the linear model significantly lacked fit for all chiral selectors, indicating either presence of quadratic of higher orders of some or all the factors that are impossible to reveal with a linear model, or the presence of other factors that needed to be included in the study via a systematic screening. In this study, the BGE, applied voltage, and temperature were not screened for relevance and were set constant (50 mM phosphate-triethanolamine, 15 kV, and 25 °C, respectively), and only pH and CD concentration were optimized. As such, the determination of a working point may be possible, yet the definition of the MODR is impossible without a proper fit of the regression model (Table 2).

Due to the importance of CEKC in the determination of the eudismic ratio and chiral purity of drugs, where the eutomer has a significantly higher effect of distomer or where the latter has undesirable side effects, many studies consider DoE a backbone of this type of studies, despite the flaws in reproducibility that seem to exist in column preparation, thus affecting the reliability of regression models [142]. Harnisch et al. [143] use a Central Composite Design (CCD) and Monte Carlo simulations (MCS) to define the DS of enantioseparation of the anticonvulsant anxiolytic prepagabalin in commercial capsules after its derivatization by dansyl chloride (Table 2).

DoE tools were also used to test method robustness prior to validation [144,145]. Using a quick Placket Burman Design (PBD), studying the effect of small, deliberate changes in CMPs around the optimum working point did not result in a significant change in CMAs, namely, resolution and migration time whose ATP were resolution ≥2.5 and migration time ≤30 min.

The calcimimetic cinacalcet was similarly the target of a similar study, where reporting enantiopurity is requested by pharmacopeial monographs and/or regulatory agencies, yet in contrast to prepagabalin, its *R*-enantiomer is the eutomer [144].

An Ishikawa fishbone diagram and Constant-Noise-Experiment (CNX) tool were used for the screening phase in this study. Even though fractional factorial designs are more common in electrophoretic and chromatographic DoE based studies, yet the CNX tool nicely identified CEKC injection, detection, capillary factors, and BGE factors, such as type and concentration of buffer, type of organic modifier, type of CD, and its degree of substitution as Constant (C). It is, in fact, common in many DoE based CEKC studies to find phosphate buffer in a range of 50–150 mM (less commonly citrate buffer), pH 2.5–6.5 (depending on pKa of analyte), and temperature in a range of 15–25 °C and 10–15 KV (Table 1), due to their major effect on EOF and electrophoretic mobilities. However, voltage and BGE characteristics (buffer pH, hydroxypropyl-γ-CD concentration, and % methanol) were considered Experimental (X) and were solely moved to the second phase of optimization. The friendly PBD was used to test method robustness prior to validation with values around the working point by Harnisch et al. [143] and Niedermeier et al. [146].

It is also possible to use this quick technique to validate the MODR per se, but here at extremes of the CMP ranges and to verify that the ATP is also fulfilled in these extreme points as reported by Pasquini et al. [144].

Even though factors affecting CEKC separations are majorly voltage, temperature, BGE ionic strength, and pH in addition to chiral selector type and concentration, it appears that a small % methanol [144] or isopropanol [147] may alter the enantioresolution, likely due to its effect on BGE viscosity and conductance.

When addressing the selection of 3-D full factorial design (FFD), CEKC analysts seem to favor face-centered CCD over CCD with axial points out of the cube space, that have a default α-value of 1.2, even though chromatography usually use this later type of CCD. Reports do not reason this selection of limited space face-centered CCD [143,145,146], yet it is well known that experimental points out of the cube space are on many occasions not practically applicable, due to instrument limitations, here probably voltage and temperature which have recommended high and low settings, respectively. It is indeed of paramount importance to select an applicable experimental domain to avoid breaking an experimental design for practical or technical reasons.

**Table 2:** Summary of DoE based CEKC methods: Full factorial Design, (FFD, full factorial design; fFD, fractional factorial design; CCD, Central Composite Design; BBD, Box-Behnken Design; MCS, Monte Carlo Simulation, PBD, Placket Burman Design; Rs, resolution; t, migration time, PhB, phosphate buffer; OFAT, One-factor-at-a time).

Application	Screening Design	Screened Factors	Optimization Design	CMPs	CQAs and ATP	Optimum Conditions	Software	Ref.
Enantioseparation of donepezil, rivastigmine, ketoconazole, itraconazole, fluconazole, sertaconazole	None	Type of CD (highly sulfated α, γ-CDs, hydroxyl propyl-β-CD, and sulfobutyl-ether-β-CD)	FFD (32)	pH, % CD	Rs; t	BGE: 50 mM phosphate-triethanolamine, 15 kV and 25 °C, pH 2.5 at low % highly sulfated-γ-CD or high % sulfobutyl-ether-β-CD	Minitab17 (USA)	[141]
Chiral purity of pregabalin as dansyl derivative	D-optimal	BGE, pH, concentration of chiral selector, voltage, temperature.	Face-centered CCD and MCS	All screened factors	Rs; t Determine impurities at ≤0.015% S-eutomer	BGE: 100 mM PhB, pH 2.5, 40 mg/mL heptakis (2,3,6-tri-*O*-methyl)-β-CDat 25 °C and 15 kV.	MODDE 11 (Sweden)	[143]
Chiral purity of cinacalcet	Ishikawa fishbone diagram and CNX tool	Type of CD: (2-carboxyethyl)-β-CD and (2-hydroxypropyl)-γ-CD	BBD and MCS	Voltage, buffer pH, % methanol, CD concentration	Rs; t Determine impurities at ≤0.1% R-eutomer	BGE: 150 mM PhB pH 2.70, 3.1 mM HPγCyD; 2.00% *v*/*v* methanol at 18 °C and 26 kV.	MODDE 10 (Sweden)	[144]
Chiral purity of dexmedetomidine	FFD	Type of CD (native α-CD, β-CD, γ-CD, neutral and charged derivatives), buffer type, addition of triethanolamine	Face-centered CCD	Voltage, temperature, buffer pH and concentration, CD concentration	Rs; t Determine impurities at 0.1% eutomer	BGE: 50 mM PhB pH 6.5, 40 mg/mL sulfated β-CD at 17 °C and 10 kV	MODDE 11 (Sweden)	[145]
Chiral purity of levomepromazine	fFD	Type of CD (charged α-CD, β-CD; γ-CD, and its neutral derivative hydroxypropyl-γ-CD). Type of buffer.	Face-centered CCD, MCS	CD concentration, buffer pH and concentration temperature, voltage	Rs; t; absence of separation of sulfoxide diastereomers. Quantify 0.1% of dextromepromazine and levomepromazine sulfoxide impurities	100 mM citric acid buffer pH 2.85, 3.6 mg/mL hydroxypropyl-γ-CD, at 15 °C and 25 kV.	MODDE 11 (Sweden)	[146]
Chiral purity of levodropropizine	OFAT	Type of CD: sulfated-α-CD; carboxymethyl-α-CD; succinyl-β-CD; Sulfated-β-CD	Face-centered CCD; MCS	CD concentration, % propan-2-ol; temperature; voltage	Rs; t; max analyses time 20 min. Quantify 0.5% of dextrodropropizine and 1-phenylpiperazine maximum analysis time of 20 min	25 mM PhB pH 7.0, 23.5 mg/mL sulfated-β-CD and 10% propan-2-ol at 16.3 °C and 16.5 kV	MODDE 12 (Sweden)	[147]
Enantiosepartion of venlafaxine	OFAT	Type of CD (neutral α-CD, β-CD, γ-CD, hydroxypropyl-β-CD, randomly methylated-β-CD, heptakis(2,6-di-*o*-methyl)-β-CD, heptakis(2,4,6-tri-*o*-methyl)-β-CD, carboxymethyl-β-CD, sulfobuthyl ether β-CD), pH	Face-centered CCD	CD concentration, BGE concentration, temperature, voltage, injection pressure	Rs; t	10 mM carboxymethyl-β-CD; pH 2.5; at 15 °C and 25 kV	Design Expert 7.0	[110]

### 3.4. Molecular Modeling Applied to Enantioseparations

As mentioned in the previous sections, CE plays a crucial role in enantiomeric discrimination. In this sense, aiming to elucidate the enantioseparation process at the molecular level, as well as the EMO, molecular modeling has proven to be an excellent auxiliary tool for the understanding of molecular recognition mechanism between selector and selectand. Besides, theoretical investigations have also been considered as powerful allies in CE experiments [148].

It is estimated that over 50% of CE enantiomeric separations occur by adding CDs to running electrolytes. Association (i.e., selector-selectand complexes with peripheral interactions) or inclusion complexes of the host-guest type are frequently studied. In special, several studies have unequivocally shown that the inclusion complex formation is not a prerequisite for chiral recognition by CDs, mainly when substituted-CDs are used, which can offer sites of interactions with selectand. Thus, the most theoretical studies in the enantioseparation field are devoted to CD host-guest inclusion complexes to comprehend the complexation mechanism and to correlate the experimental results with the mode of inclusion of the guest molecule (analyte) into the CD host (chiral selector). Furthermore, molecular modeling has been used to explain not only selector-selectand interaction, but also the migration phenomena observed in CE [139,149]. Previous works presented the elucidation of chiral recognition mechanisms in separation sciences by various selectors. Scriba reviewed the main contributions in the literature about the enantiorecognition mechanisms of main chiral selectors, i.e., polysaccharide, cyclodextrins, macrocyclic glycopeptides, donor-acceptor-type selectors, chiral ion-exchangers, chiral ligand-exchangers as well chiral micelles [150,151]. Peluso et al. [152] presented the progress in docking and molecular dynamics (MD) simulations based on selector type. In this context, this review section illustrates the combined approach using CE and molecular modeling for understanding the chiral recognition mechanism using CDs.

From a structural point of view, CD complexes differ from each other in terms of the spatial and geometric arrangement, due to the chemical nature, strength, and number of interactions established. Under an energetic point of view, they differ in terms of the free energy of Gibbs (ΔG) formation variation, and the magnitude of the ΔG values, fundamentally depend on the enthalpic (ΔH) and entropic (−TΔS) contributions associated with each complex formation, involving each of the enantiomers (ΔG = ΔH − TΔS) [42,153]. In this sense, theoretical/computational studies can lead to a reliable prediction of EMO through structural, topological, and energetic analyses of the CD complexes, which reveal details on the intermolecular forces, as well as electronic and thermodynamic properties.

The computational studies combined or not with CE experiments mostly report using classical theoretical methods, such as Molecular Dynamics (MD) and Molecular Docking (MDoc). However, works using Semiempirical (SE) methods, hybrid techniques, as the Quantum Mechanics/Molecular Mechanics (QM-MM), as well as pure quantum-mechanical approach, as the Density Functional Theory (DFT) can also be found. Table 3 contains an overview of theoretical works, along with experimental data, from the years January 2016 to March 2021 that deals with the study of chiral discrimination using CDs. As can be seen, several research groups have dedicated themselves to study the chiral discrimination mechanism by CDs, aiming to predict or confirm EMO.

It is important to mention that the selector–selectand interaction is only one part of the entire separation process in CE. Another part also important is associated with the dynamics (mobilities) involved in chiral discrimination. In this sense, MD simulations have been used in an attempt to explain, at the molecular level, the mechanisms of chiral separation and EMO.

In 2017, Salgado et al. [154], combined NMR spectroscopy experiments with MD calculations, to obtain structural and energetic information on the interactions between clenbuterol enantiomers with β-CD and heptakis-(2,3-di-*O*-acetyl)-β-CD. The binding energies between each of the enantiomers and the evaluated CDs were calculated, in addition to the different contributions to total binding energy, divided into van der Waals, coulombic, desolvation, and nonpolar energy. In view of the numerical data, the authors showed that the energies computed via MD, were proportional to the experimental equilibrium bond constants.

**Table 3:** List of representative works published between 2016 and March 2021 that deal with the study of chiral discrimination by CDs for EMO prediction or confirmation in CEKC.

Analyte	CDs	Separation Conditions	Theoretical Methodology	Evidence of Interactions	Ref.
Tramadol	Sulfated-β-CD, carboxymethyl-β-CD	Phosphate buffer 125 mM (pH 10), 20 kV, 30 mbar/4 s, 10 °C, detection at 195 nm	Semiempirical PM3/DFT (B3LYP/6-31G+(d,p))/water solvent (PCM)	Hydrogen bonds	[44]
Bupropion and hydroxybupropion	sulfated-β-CD	BGE phosphate 75 mM pH 7.0, 15 kV, 30 mbar/4 s, 15 °C, detection at 210 nm	Molecular dynamics simulations with AMBER/DFT force field (B97D(6-31G(d,p))/water solvent (SMD)	Hydrogen bonds	[155]
Clenpenterol	β-CD/heptakis(2,3-di-*O*-acetyl)-β-CD	Phosphate buffer 50 mM pH 2.0	Molecular dynamics simulations (100 ns trajectories) using amber force field	Van der Waals interactions, hydrogen bonds, and desolvation energy	[154]
Quinurenine	α-CD, mono-6A-deoxy-6-(1-allylimidazolium)-β-CD chloride, and their mixture	Borax buffer 50 mM pH 9.0, 15 kV, 50 mbar/5 s, 25 °C, detection at 226 nm	Molecular docking/molecular mechanics MM2	Hydrogen bonds	[156]
Medetomidine	β-CD, γ-CD, Sulfated-β-CD and highly sulfated-β-CD	Phosphate buffer 50 mM pH 2.5, 20/−20 kV	Molecular dynamics simulations in water (TIP3P/12 Å, 100 ns trajectories) and use of amber force field	Hydrogen bonds	[157]
Citalopram	Carboxymethyl-β-CD,	Phosphate buffer 25 mM pH 7.0, 15 kV, 50 mbar/1 s, 17.5 °C, detection at 240 nm	Semiempirical RM1/DFT M06-2X-D3(6-31G**)/water solvent by SM8 method	Details of structural analysis were not provided	[158]
Asenapine	β-CD	TRIS-acetate buffer 160 mM pH 3.5, 15 kV, 50 mbar/4 s, 20 °C	Semiempirical PM3/DFT PBE def2-SVP/water solvent by COSMO method	Hydrogen bonds	[159]
Sutezolid	heptakis-(2,3-diacetyl-6-sulfo)-β-CD heptakis-(2,3-dimethyl-6-sulfo)-β-cyclodextrin	NACE buffer (methanol: acetonitrile: trifluoroacetic acid), 25 kV, 0.5 psi/5 s, 22 °C, detection at 200 and 258 nm	DFT/B3LYP(6-31G*)/water solvent (TIP3/14 Å) (Amber 14 molecular dynamics simulations (MM-GBSA/MMPBSA, trajectories up to 500 ns)	Hydrogen bonds	[160]
Oxybutynin, clenbuterol, salbutamol and peneiclidine	heptakis-(2,3-diacetyl-6-sulfo)-β-CD	BGE TRIS-H_3_PO_4_ 50 mM pH 2.5, 10 kV, 10 mbar/3 s, detection at 210 nm	Molecular Mechanics Powell Method (Strength Field Tripos)/Molecular Dynamics Simulations with LGA Algorithm	Hydrogen bonding, nonclassical hydrogen bonding and π-S	[161]
Rasagiline	Sulfobutyl-ether-β-CD	Glycine-HCl buffer 50 mM pH 2.0, 12 kV, 25 mbar/2 s, 35 °C, detection at 200 nm	Molecular docking simulations/implicit solvency of Generalized Born	Van der Waals interactions, hydrogen bonds, and type-π	[162]
Ofloxacin	Methylated-β-CD	Phosphate buffer 50 mM pH 3.1, 20 kV, 50 mbar/5 s, 20 °C, detection at 276 nm	Molecular Mechanics MM2	Van der Waals interactions and electrostatic interactions (interactions of loads, dipoles, and quadrupoles)	[163]
Bronpheniramine, chlorpheniramine and pheniramine	heptakis {2,6-di-*O*-[3-(1,3-dicarboxylpropylamino)-2-hydroxypropyl]}-β-CD	Phosphate buffer 120 mM pH 2.5–4.0, 20 kV, 20 psi, 20 °C, detection at 210 nm	Hybrid method ONIOM2: Semiempirical PM3/DFT (B3LYP/6-31G(d,p))	Van der Waals interactions and electrostatic interactions	[164]
Acebutelol	heptakis-(2,3-diacetyl-6-sulfo)-β-CD/heptakis(2,3-di-*O*-methyl-6-*O*-sulfo)-β-CD	Phosphate buffer 100 mM pH 3.0 and ammonium format 0.75 mM, 25 kV, 50 mbar/3 s, 15 °C, detection at 230 nm.	Molecular dynamics simulations with general amber force field with AM1-BCC load. For purpose solvent model IEFPCM (aqueous solution and methanol solution)	Hydrophobic effect and hydrogen bonds	[165]
Amlodipine	carboxymethyl-β-CD	BGE TRIS-H_3_PO_4_ 20 mM pH 3.5, 20 kV, 50 mbar/5 s, 20 °C, detection at 237 nm.	Molecular mechanics (MM2) and molecular dynamics simulations/Semiempirical method PM3	Hydrogen bond, dipole-dipole, and π-π.	[90]
Terbutalin	heptakis {2,6-di-*O*-[2-hydroxy-3-(sulfoamino)propoxy]}-β-CD	Phosphate buffer 60 mM pH 2.5, 10 kV, 0.5 psi/5 s, detection at 210 nm.	Hybrid method ONIOM2: Semiempirical PM3/DFT (B3LYP/6-31G(d,p))	Electrostatic interactions and hydrogen bonding	[166]

Although most of the theoretical calculations are performed via MD simulations, some precaution is necessary. One aspect that must be considered in MD simulations is related to the selector-selectand interactions. It is known that multiple long- and short-range interactions are involved in the chiral separations, but not all these forces are enantioselective. In this sense, MD simulations must also be used cautiously, especially in the studies aimed to explain EMO, which must also consider systems that have a reverse migration order, due to concentration, pH, or other variables [55,149]. Moreover, all possible conditions must be inserted and considered in the MD simulations, for example, selector and selectand charges, force field, nature of the electrolytes, among others, along with experimental validation of studied CD complexes [167,168].

On the other hand, the use of quantum mechanical high-level calculations, such as DFT, to study the enantioseparation process has risen, especially in recent years. DFT is fundamentally a more precise theoretical methodology than those based on molecular mechanics, since it is more reliable and efficient in describing the energy and electronic structure of chemical systems and complexes. In 2019, Michalska et al. [160], revealed through DFT calculations, structural details about separating sutezolid enantiomers with heptakis-(2,3-dimethyl-6-sulfo)-β-CD, having been demonstrated the significant influence of the inclusion of thiomorpholine rings in the CD cavity and hydrogen bonds, in the formation and stability of the formed diastereoisomeric complexes. Through the enthalpy variation values (ΔH) obtained, the authors demonstrated that according to the experimental conditions used, protonated sutezolid favors forming a more stable complex.

In 2017, Cecilio et al. [44] conducted a computational chiral discrimination study of opioid tramadol with sulfated-β-CD and carboxymethyl-β-CD. Using a sequential methodology, which involved molecular dynamics simulations, semiempirical calculations (PM3), and DFT at the B3LYP/6-31G(d,p) level, it was possible to explain the EMO of tramadol enantiomers through a structural and energetic association complexes analysis, i.e., complexes formed between functional groups enantiomers with active sites outside the CD cavity. The results showed that forming shorter and stronger hydrogen bonds between (R)-tramadol enantiomer and CDs, led to more stable values of ΔE, which allowed the experimental migration order prediction. Along with the theoretical results, fractions of pure enantiomers were obtained and injected into the electrophoretic system, whose migration times by experimental studies corroborated with theoretical predictions.

In 2019, Pires et al. [155] developed an enantioselective method for simultaneous separation of bupropion and hydroxyibrupropion using sulfated-β-CD as a chiral selector. After optimizing the experimental conditions that influence the separation, such as concentration and pH of BGE and concentration and type of CD, it was possible to obtain an electropherogram with optimized conditions. The EMO was elucidated using DFT calculations at B97D/6-31G(d,p) level. One interaction of hydrogen bond at a distance of 2 Å was enough to (S)-bupropion@sulfated-β-CD complex to show greater stability than (R)-bupropion@sulfated-β-CD. Similar behavior was also observed for the hydroxybupropion@sulfated-β-CD complexes. Notably, the theoretical study contribution to the experiment proved to be very relevant, since it was possible to determine the EMO.

**Figure 9:**
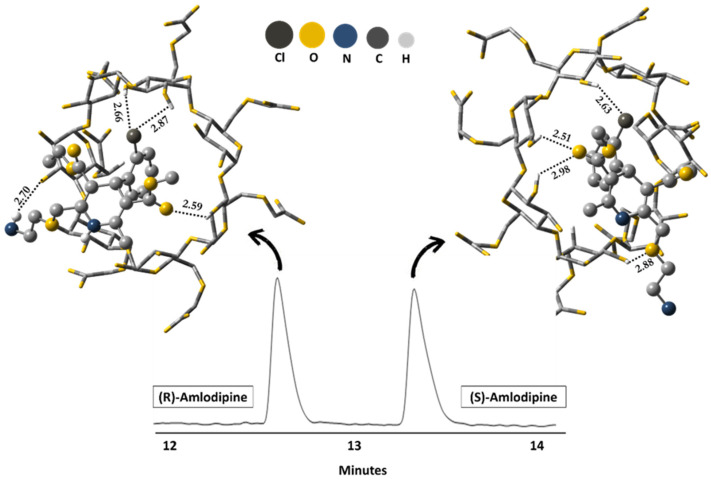
Electropherogram related to conditions optimized for enantioseparation of amlodipine and diastereoisomeric complexes obtained via DFT calculations (B97D/6-31G(d,p)) in the aqueous phase. Experimental conditions: 5.5 mg/mL carboxymethyl-β-CD in 50 mM BGE phosphate (NaH_2_PO_4_) pH 4.0; uncoated fused-silica capillary 75 µm id × 62.5 cm (71 cm to the detector); hydrodynamic injection using 40 mbar × 4 s; separation voltage at 15 kV; UV detection at 195 nm. Source: Elaborated by the authors themselves (our unpublished results).

Currently, our research group is involving the enantioselective separation of the amlodipine using carboxymethyl-β-CD as a chiral selector. Figure 9 shows an electropherogram with optimized conditions, which are similar to those proposed by Hancu et al. [169]. In this condition, (*R*)-amlodipine enantiomer presented a migration time shorter than the (*S*)-amlodipine enantiomer. Combining the experiments with PM3/DFT calculations (B97D/6-31G(d,p)), it was possible to explain at a molecular level the diastereoisomeric complex (*S*)-amlodipine@carboxymethyl-β-CD (ΔG = −15.8 kcal/mol) formation, more stable than (*R*)-amlodipine@carboxymethyl-β-CD (ΔG = −12.7 kcal/mol), due to the formation of stronger hydrogen bonds by including the (*S*)-amlodipine enantiomer on the primary face of the CD.

Despite the rapid increase in the number of papers involving DFT calculations in the last years, semiempirical methods still attract a great deal of attention owing to their lower computational demands. For example, semiempirical methods, such as PM3 and PM6, have been found to give good estimates of molecular properties than ab initio and DFT methods at even lower computational cost, making it an attractive method for the description of inclusion complexes [170]. Budău et al. [158] studied, via semiempirical calculations RM1, the formation of the host-guest complexes formed between citalopram and carboxymethyl-β-CD. The authors correlated EMO, with the formation of a more stable complex involving the enantiomer with longer migration time, i.e., (R)-citalopram@carboxymethyl-β-CD (ΔE = −67.75 kJ/mol), than the antipode (*S*)-citalopram@carboxymethyl-β-CD (ΔE = −48.32 kJ/mol).

Several papers published in the last six years, presented in this review, show great potential of molecular modeling for understanding enantioselective recognition mechanisms. A systematic theoretical analysis, when used in combination or not with CE experiments, can provide a good perspective on the enantioseparation process, serving as a valuable auxiliary method for studying chiral recognition mechanisms. Finally, efforts in the computational chemistry field are continuously required—since the mechanistic aspects of selector-selectand interactions, at the molecular level, are still, in many cases, not fully understood.

## Figures and Tables

**Table 1:** List of representative works published between 2016 and March 2021 dealing with the application of CEKC in enantiomeric separation and determination of enantiomeric compounds from different fields.

Analyte	Matrix	Separation Conditions	Detection	LOD/LOQ	Ref.
Cathinone derivatives	Human hair	Preconcentration by solid phase extraction, fused silica capillary of 50 µm i.d. and 80 cm total length, separation voltage of 35 kV, BGE of 80 mM disodium phosphate at pH 2.5, β-CD as CS	DAD at 200 nm	0.02 ng/mg 0.2 ng/mg	[94]
Proteinogenic amino acids	Cerebrospinal fluid	Derivatization by 9-fluorenylmethyl chloroformate fused silica capillary of 50 µm i.d. with a length of 70 cm to the detector and 80 cm of total length, separation voltage of 35 kV, BGE of 50 mM ammonium bicarbonate at pH 8 containing 15% (*v*/*v*) isopropanol and 10 mM β-CD, Sheath liquid interfacing of propan-2-ol:water: 1 M ammonium bicarbonate (50:50:1, *v*/*v*/*v*) at a flow rate of 3 L/min, and a nebulizer gas pressure of 2 psi	ESI-MS	0.9 µM -	[95]
Tedizolid	Pharmaceutical formulation	Fused silica capillary of 45 cm (effective length 35 cm) × 25 µm i.d., separation voltage of 12 kV, BGE of 37.5 mM heptakis-(2,3-diacetyl6-sulfo)-β-CD dissolved in 50 mM formic buffer pH 4.0 with the addition of acetonitrile (81.4:18.6, *v*/*v*)	DAD at 200 nm	- -	[96]
Radezolid	Pharmaceutical preparation	Fused silica capillary of 45 total length and 35 cm effective length and 25 µm i.d., separation voltage −28 kV, BGE of 40 mM heptakis(2,3-di-*O*-methyl-6-sulfo)-β-CD dissolved in 50 mM phosphate buffer pH 2.5	DAD at 265 nm	- -	[97]
Methadone	Exhaled breath condensate	Fused silica capillary of 50 cm length, 41.5 cm effective length, and 50 µm i.d., separation voltage of 25 kV, BGE of 150 mM phosphoric acid-tetraethylammonium at pH 2.5 containing 30% (*v*/*v*) methanol and 0.8% (*w*/*v*) carboxymethyl-β-CD	DAD at 200 nm	- 0.15 µg/mL	[98]
Colchicine	Pharmaceutical preparation	Fused silica capillary 58.5 cm (50 cm effective length) × 50 μm i.d., separation voltage of 20 kV, 50 mM or 25 mM borate buffer pH 9.0 using succinyl-γ-cyclodextrin or sulfated-γ-CD	UV at 243 nm	0.3 mg/mL 1 mg/mL	[99]
Praziquantel	Pharmaceutical preparation	Fused silica capillary of 50 µm i.d., 48.5 cm total and 40 cm effective length, separation voltage of 15 kV, BGE of 50 mM phosphate buffer pH 2.0, supplied with 15 mM sulfated-β-CD	DAD at 210 nm	0.75 µg/mL 2.0 µg/mL	[100]
Glycopyrrolate	Rat plasma	Online preconcentration by cation-selective exhaustive injection-sweeping, fused silica capillary (40.2 cm × 75 μm), separation voltage of −20 kV, BGE 30 mM phosphate solution at pH 2.0 containing 20 mg/mL sulfated-β-CD and 5% acetonitrile	DAD at 200 nm	2.0 ng/mL 0.0625 µg/mL	[101]
Brompheniramine	Rat plasma	Online preconcentration by cation-selective exhaustive injection and sweeping, fused silica capillary of the total length of 50 cm (effective length 40 cm) × 50 µm i.d., separation voltage of −20 kV, BGE in 50 mM phosphate buffer pH 3.5, containing 10% (*v*/*v*) acetonitrile and 30 mg/mL sulfated-β-CD	UV at 210 nm	- 0.01 µg/mL	[102]
Ivabradine	Pharmaceutical formulation	Fused silica capillary of 58.5 cm (50 cm to the detector window) × 50 µm i.d., separation voltage of −30 kV, BGE of 5 mM tetrabutylammonium-aspartic acid in 50 mM formate buffer pH 2.0 containing 4 mM sulfated-γ-CD	UV at 200 nm	0.22 and 0.28 µg/mL 0.73 and 0.93 µg/mL	[103]
Lansoprazole and rabeprazole	Pharmaceutical preparations	Fused silica capillary of 48 cm total, and 40 cm effective length and 50 μm i.d., separation voltage of +20 kV, BGE for lansoprazole: 25 mM phosphate buffer pH 7, 10 mM sulfobutyl-ether-β-CD/20mM γ-CD, +20 kV voltage; BGE for rabeprazole: 25 mM phosphate buffer pH 7, 15 mM sulfobutyl-ether-β-CD/30 mM γ-CD	UV at 210 nm	2 and 2 µg/mL 6 and 6 µg/mL	[88]
Pheniramine	Rat plasma	Online preconcentration by large volume sample stacking and sweeping, fussed silica capillary of a total length of 50 cm (effective length 40 cm) × 50 μm i.d., separation voltage of −20 kV, BGE of 30 mM phosphate buffer at pH 3.0 with 30 mg/mL sulfated-β-CD	UV at 262 nm	- 10 ng/mL	[104]
Phenothiazines	Urine sample	Preconcentration by solid phase extraction, fused silica capillary of 75 µm i.d. and 365 µm o.d., separation voltage from 10 to 12 kV, BGE of 75 mM phosphate buffer pH 3.0 and 0.9% poly (diallyldimethylammonium chloride), hydroxypropyl-γ-CD as a CS.	UV at 254 nm	2.1 to 6.3 nM-	[105]
Six phenoxy acid herbicides (Fenoprop 1, Fenoprop 2, Mecoprop 1, Mecoprop 2, Dichlorprop 1, Dichlorprop 2	Mixture of herbicides	Fused silica capillary of 58.5 cm total length and 50 cm length to the detector and 50 µm i.d., separation voltage of 25 kV, BGE of 50 mM phosphate buffer pH 7.0, dual CD (4 mM hydroxyl--β-CD and 16 mM heptakis(2,3,6-tri-*O*-methyl)-β-CD.	DAD at 200 nm for mecoprop, chlorprop, and 210 nm for fenoprop	- -	[106]
Homocysteine and cysteine	Stock standard solutions in borate buffer	Derivatization by 9-fluorenylmethyl chloroformate, fused silica capillary of 58.5 cm total length and 50 cm effective length and 50 µm i.d., separation voltage 20 kV, BGE for homocysteine: 2 mM γ-CD +5 mM L-Carnitine C1NTf2 in borate buffer pH 9.0, BGE for Cysteine: 2 mM γ-CD +5 mM L-CarnitineC1Lac in phosphate buffer pH 7.0	DAD at 210 nm	- -	[107]
Amlodipine	Pharmaceutical formulation	Fused silica capillary of 48 cm length (40 cm effective length) ×50 μm i.d., separation voltage of 25 kV, BGE of 25 mM phosphate buffer pH 9.0, 15 mM carboxymethyl-β-CD	UV at 230 nm	S 0.27 and R 0.32 µg/mL S 0.8 and R 0.96 µg/mL	[108]
L/D-Asp, L/D-Glu, and L/D-Ser	Bone cell lines (murine osteocytes and osteoblast	Derivatization by 4-fluoro-7-nitro-2,1,3-benzoxadiazole, capillary fused silica with 75 μm i.d. and a total length of 60 cm, separation voltage of 30 kV, BGE 137.5 mM borate buffer pH 10.25 and 12.5 mM β-CDs	LIF at λ_ex_ = 488 nm and λ_em_ = 522 nm	0.25 µmol/L	[109]
Venlafaxine	Pharmaceutical preparations	Fused silica capillary of 30 cm length (effective length 22 cm) × 50 µm, separation voltage of 25 kV, BGE of 25 mM phosphate buffer pH 2.5, 10 mM carboxymethyl-β-CD	UV at 230 nm	0.07 and 0.06 mg/mL 0.21 and 0.18 mg/mL	[110]
Methylparaben, ethylparaben, propylparaben, butylparaben, isobutylparaben, sorbic acid, benzoic acid, p-hydroxybenzoic acid	Pharmaceutical preparations	Online preconcentration by large volume sample stacking, fused silica capillary of 75 µm i.d. × 50 cm length, separation voltage 25 kV, BGE of 25 mM tetraborate pH 9.3 and α-CD	UV at 195 nm for ethylparaben, benzoic acid, and p-hydroxybenzoic acid, at 296 nm for methylparaben, propylparaben, butylparaben, and isobutylparaben, at 254 nm for sorbic acid at 254 nm.	0.8 to 5 ng/mL 3 to 16 ng/mL	[111]
L-Panthenol dexapanthenol	Pharmaceutical and cosmetic formulations	Fused silica capillary of a total length of 58.5 cm (50 cm effective length) and 50 µm i.d., separation voltage of 30 kV, BGE of 25 mM (2-carboxyethyl)-β-CD in 100 mM borate buffer pH 9.0	UV at 205 nm	1.0 and 4.0 mg/L 3.3 and 13.3 mg/L	[112]

## Data Availability

The data presented in this study are available within the article or on request from the corresponding author.
